# The Impact of lncRNAs and miRNAs on Apoptosis in Lung Cancer

**DOI:** 10.3389/fonc.2021.714795

**Published:** 2021-07-21

**Authors:** Soudeh Ghafouri-Fard, Amin Aghabalazade, Hamed Shoorei, Jamal Majidpoor, Mohammad Taheri, Majid Mokhtari

**Affiliations:** ^1^ Department of Medical Genetics, School of Medicine, Shahid Beheshti University of Medical Sciences, Tehran, Iran; ^2^ Department of Pharmacology, Tabriz University of Medical Sciences, Tabriz, Iran; ^3^ Department of Anatomical Sciences, Faculty of Medicine, Birjand University of Medical Sciences, Birjand, Iran; ^4^ Department of Anatomical Sciences, School of Medicine, Gonabad University of Medical Sciences, Gonabad, Iran; ^5^ Skull Base Research Center, Shahid Beheshti University of Medical Sciences, Tehran, Iran; ^6^ Critical Care Quality improvement Research Center, Loghman Hakim Hospital, Shahid Beheshti University of Medical Sciences, Tehran, Iran

**Keywords:** lncRNA, miRNA, apoptosis, lung cancer, expression

## Abstract

Apoptosis is a coordinated cellular process that occurs in several physiological situations. Dysregulation of apoptosis has been documented in numerous pathological situations, particularly cancer. Non-coding RNAs regulate apoptosis *via* different mechanisms. Lung cancer is among neoplastic conditions in which the role of non-coding RNAs in the regulation of apoptosis has been investigated. Non-coding RNAs that regulate apoptosis in lung cancer have functional interactions with PI3K/Akt, PTEN, GSK-3β, NF-κB, Bcl-2, Bax, p53, mTOR and other important cancer-related pathways. Globally, over-expression of apoptosis-blocking non-coding RNAs has been associated with poor prognosis of patients, while apoptosis-promoting ones have the opposite effect. In the current paper, we describe the impact of lncRNAs and miRNAs on cell apoptosis in lung cancer.

## Introduction

Apoptosis is a well-organized and coordinated cellular process that happens in several physiological situations. Aberrant regulation of apoptosis has also been documented in numerous pathological situations, particularly cancer. In fact, cancer is one of the circumstances where this process is reduced, leading to evolution of malignant cells that will not perish. Apoptosis is regulated by a complex mechanism involving numerous pathways. Deficiencies in apoptotic pathways lead to malignant transformation of cells, enhancement of metastasis and induction of resistance to chemotherapy/radiotherapy. Meanwhile, apoptosis has been considered as a target of several anticancer modalities (1). Both intracellular and extracellular stimuli can regulate apoptosis. This process is described by morphological alterations in the cells including fragmentation and condensation of the nuclear compartment, permeabilization of the outer membrane of mitochondria, membrane blebbing, cell shrinkage and finally formation of apoptotic bodies (2). Two extrinsic and intrinsic pathways are involved in the induction of cell apoptosis. While the extrinsic pathway is stimulated by death receptors, namely Fas, TNF receptors and TRAILs, the intrinsic pathway is initiated by DNA damage, energy starvation and hypoxia, which can dephosphorylate and cleave pro-apoptotic proteins, resulting in their recruitment in the mitochondria (3). Both pro-apoptotic and anti-apoptotic members of the Bcl-2 family proteins regulate intrinsic apoptotic pathway (4).

Recent studies have shown that non-coding RNAs (ncRNAs) have an important regulatory role on induction of apoptosis. In fact, regulation of cell apoptosis is the main route of function of many of these transcripts in the carcinogenic events (5). This group of transcripts has several types, two of them i.e. long non-coding RNAs (lncRNAs) and microRNAs (miRNAs) have attained more attention in cancer biology. LncRNAs have typically sizes more than 200 nucleotides and are transcribed by RNA polymerase II, except for few cases do not harbor open reading frame and translation-termination region, yet, lncRNAs can be spliced, 5’-capped and get polyadenylated tails. Their specific three-dimensional conformation permits them to interact with several classes of biomolecules including proteins, DNA or RNA. These interactions are framed through base pairing or construction of network (6). LncRNAs partake in regulation of gene expression, differentiation of cells and alteration of chromatin structure (6).

**Figure 1:**
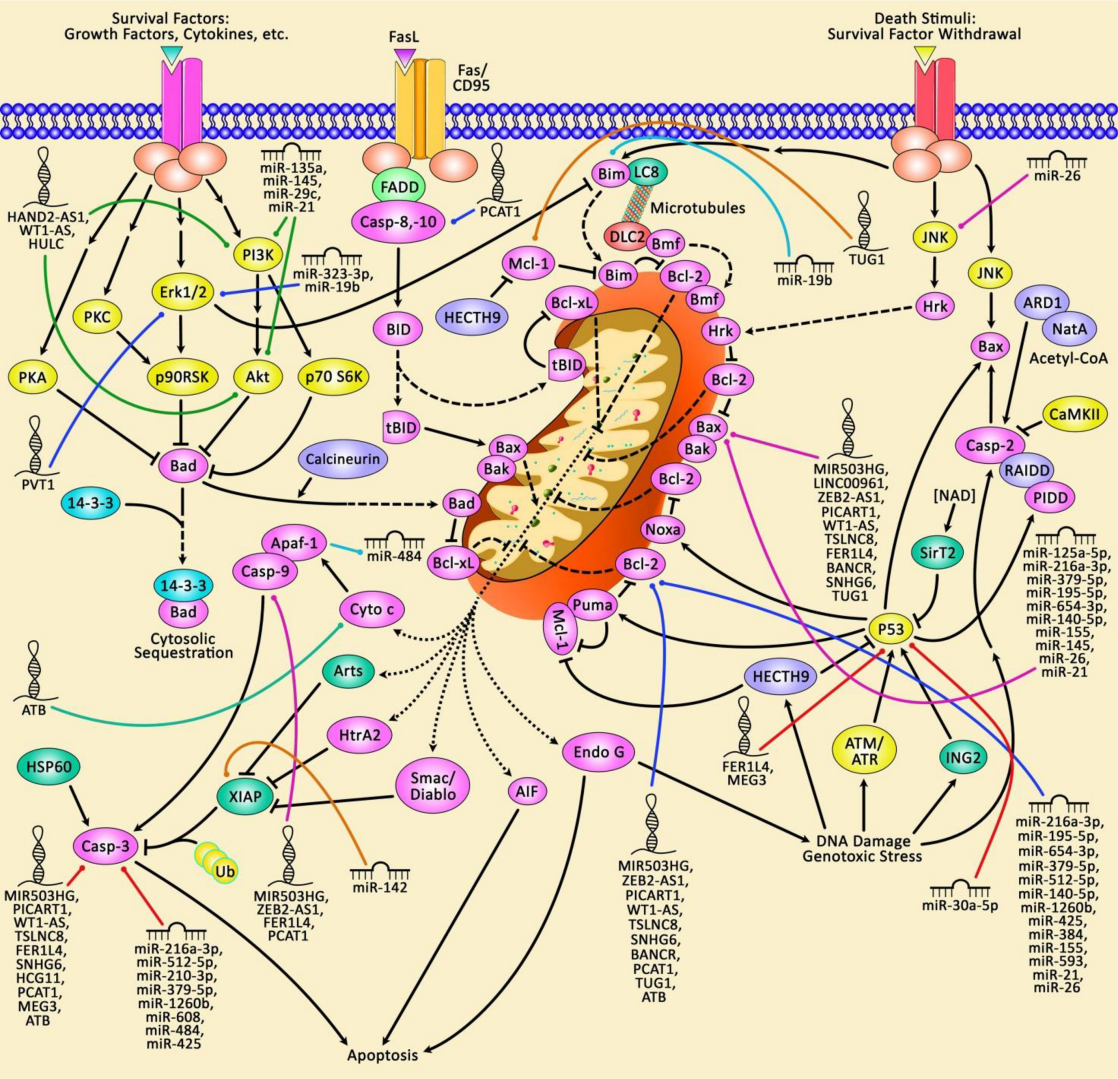
A schematic representation of the role of non-coding RNAs in triggering the mitochondrial pathway of apoptosis in human lung cancer. The Bcl-2 family of proteins could play an effective role in modulating apoptosis *via* regulating mitochondrial cascade. The anti-apoptotic proteins Bcl-2 and Bcl-xL are located in the exterior part of mitochondrial wall and can suppress cytochrome c release. The pro-apoptotic Bcl-2 proteins Bax, Bad, Bim, and Bid could be located in the cytosol but may be transferred to mitochondria following induction of death signaling pathway, where they could elevate the release of cytochrome c (8, 9). The mitochondrial cascade of apoptosis could be considered as the most commonly deregulated form of cell death in a variety of human cancers. Furthermore, aberrant expression of various non-coding RNAs could have a crucial part in dysregulating the mitochondrial pathway of apoptosis in lung cancer.

miRNAs have been shown to regulate expression of a high proportion of human genes. They mainly target 3’ UTR of genes to suppress their expression or degrade the corresponding RNAs. Several aspects of cell functioning including apoptosis is regulated by miRNAs (7). **Figure 1** illustrates that aberrant expression of various ncRNAs could contribute in modulation of the mitochondrial pathway of apoptosis in the context of lung cancer.

In the current paper, we describe the impact of lncRNAs and miRNAs on cell apoptosis in lung cancer.

## miRNAs and Apoptosis in Lung Cancer

**Figure 2:**
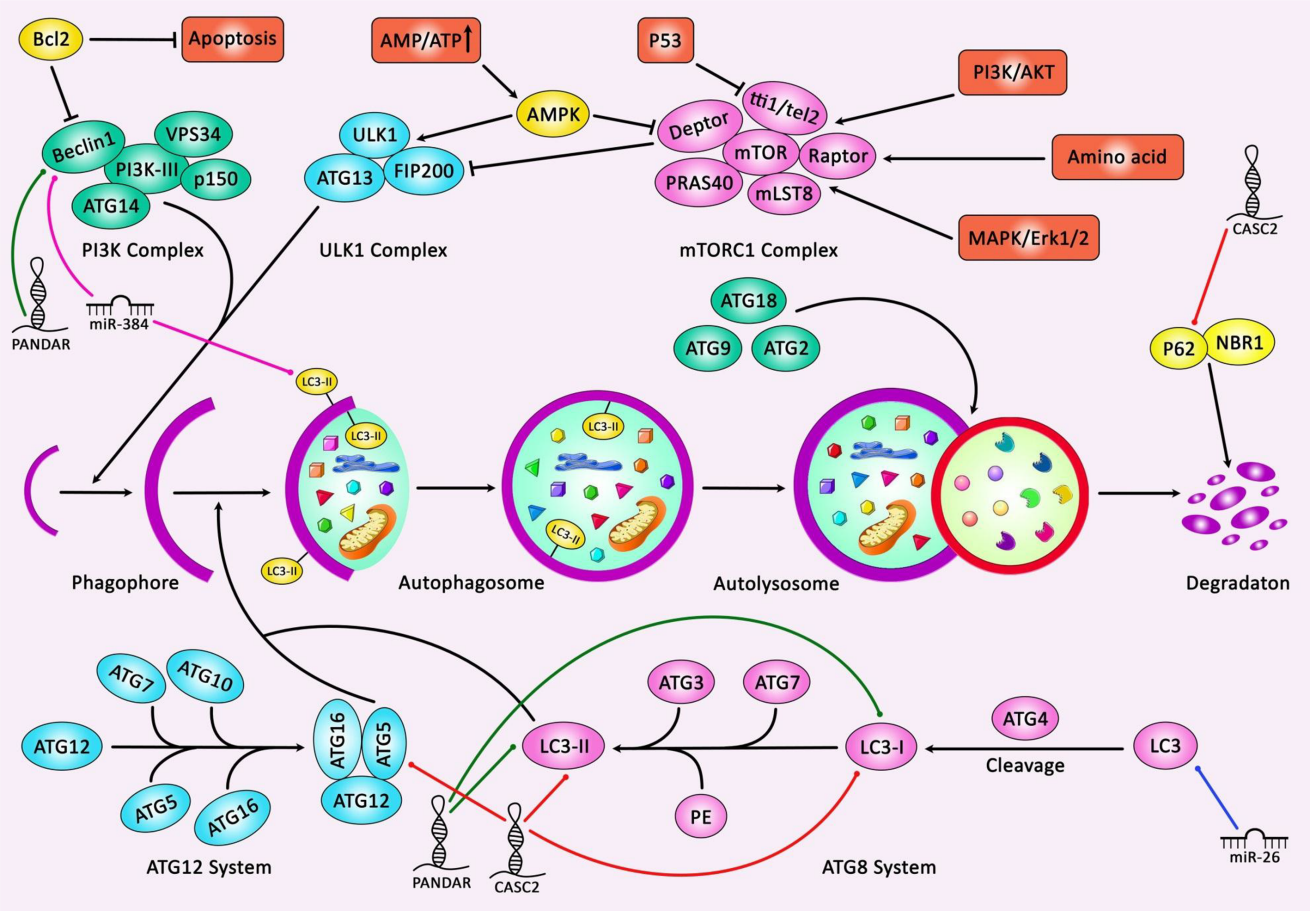
A schematic summary of the role of various non-coding RNAs in modulating the process of autophagy in human lung cancer. Several non-coding RNAs affect lung cancer progression through modulating autophagy and apoptosis cascades in human lung cancer cells. As an illustration, overexpression of lncRNA PANDAR as a tumor suppressor *via* directly targeting Beclin-1, LC3-I and LC3-II could activate both autophagy and apoptosis cascades, and thereby suppressing progression of lung cancer (14). In addition, lncRNA CASC2 could suppress autophagy and enhance apoptosis pathway in non-small cell lung cancer cells through modulating the miR-214/TRIM16 axis. Moreover, p62 expression level was significantly elevated but Atg-5 expression and the ratio of LC3-II/LC3-I were considerably reduced in the CASC2-overexpressing cells (15).

Suppression of PI3K/AKT pathway in EGFR mutant lung cancer cells has led to dysregulation of 17 miRNAs among them have been members of the miR-17~ 92 cluster. These miRNAs function in a coordinated manner to increase the activity of the EGFR cascade. Suppression of miR-19b expression in EGFR mutant lung cancer cells has led to re phosphorylation of ERK, AKT and STAT and effector proteins. Consistently, it has resulted in enhancement of apoptosis, while reduction of cell cycle progression, colony formation and migration. Administration of gefitinib along with miR-19b antagonism has decreased migration and colony formation in a synergistic manner implying the cooperation between EGFR and miR-19b in the regulation of oncogenesis. PPP2R5E and BCL2L11 have been recognized as main targets of miR-19b, through their inhibition, miR-19b regulates cell proliferation and resistance to apoptosis, respectively (10). miR-21 is another miRNA that regulates apoptosis of lung cancer cells *via* influencing the PI3K/Akt/NF-κB signaling pathway. Inhibition of miR-21 has enhanced apoptosis *via* this route. ASPP2 has been recognized as the target of miR-21 in NSCLC cells. miR-21 silencing has also inhibited migration, invasion, and epithelial-mesenchymal transition (EMT). Besides, miR-21 inhibition has stimulated cell apoptosis through caspase dependent route. Taken together, miR-21 silencing can induce cell apoptosis *via* reducing activity of the PI3K/Akt/NF-κB signaling (11). miR-24 is another oncogenic miRNA which is up-regulated in lung cancer tissues, particularly in high grade and large-sized tumors. Consistently, higher expression of miR-21 predicts lower overall survival (OS) of patients. Functionally, miR-24 enhances the viability, proliferation and cell cycle transition, while inhibiting cell apoptosis through binding with MAPK7 (12). miR-26 is a down-regulated miRNA in lung cancer cells. Forded over-expression of miR-26 induces cell apoptosis and enhances activity of caspase-3 and caspase-9. On the other hand, miR-26 silencing has increased levels of LC3 protein and the autophagy-associated genes in lung cancer cells. Besides, miR-26 has been shown to influence apoptosis and autophagy through suppressing expression of TGF-β in a JNK dependent route. Besides, miR-26 has been reported to affect the endoplasmic reticulum stress (ERS) signaling pathway (13). **Figure 2** represents the role of several ncRNAs in regulating autophagy cascade in human lung cancer.

**Table 1** shows the list of miRNAs that regulate apoptosis in lung cancer.

**Table 1:** miRNAs regulating apoptosis in lung cancer.

miR	Sample	Cell line	Target/pathway	Function	Ref
miR-19b	–	PC9, PC9ER, HCC827	Akt, ERK1/2, PTEN, GSK-3β, STAT3, PPP2R5E, BCL2L11	miR-19b *via* targeting PP2A and BIM through the EGFR signaling pathway could enhance apoptosis in NSCLC.	(10)
miR-21	Mice	HBE, A549	PI3K/Akt, NF-κB, Bcl-2, Bax, P65, Ikkβ, ASPP2, E-cadherin, N-cadherin, Vimentin	miR-21 *via* inhibiting the PI3K/Akt/NF-κB pathway could induce apoptosis in NSCLC.	(11)
miR-24	Human	BEAS-2B, A549, H292, H1703	MAPK7	miR-24 by targeting MAPK7 could promote apoptosis in LC.	(12)
miR-26	Human	A549, H1703, 801D	TGF-β1/JNK, Bcl-2, Bax, LC3	miR-26 *via* suppressing the TGF-β1/JNK pathway could induce apoptosis in NSCLC.	(13)
miR-29c	Human	A549, NCI-H1299, H1650	VEGFA, PI3K, Akt	miR-29c *via* targeting VEGFA could promote apoptosis in NSCLC.	(16)
miR-30a	Human	A549	MEF2D, Caspase-3	Knockdown of miR-30a *via* targeting MEF2D could promote apoptosis in LC.	(17)
miR‐30a‐5p	Human	A549, H1299, H460	SOX4, p53	miR‐30a‐5p by targeting SOX4 could mediate apoptosis in NSCLC.	(18)
miR-34b	Human	A549	YAF2, p-Jak2, STAT3, MMP2, Caspase-3	miR-34b *via* targeting YAF2 could promote apoptosis in NSCLC.	(19)
miR‐34b‐3p	Human	BEAS‐2B, A549, H1299	CDK4	miR‐34b‐3p *via* targeting CDK4 could repress apoptosis in NSCLC.	(20)
miR-106b-5p	Human	16HBE, H1299, SKMES1, A549, H358, SPCA1	BTG3	miR-106b-5p *via* regulating BTG3 could inhibit apoptosis in NSCLC.	(21)
miR-124	Human	BEAS-2B, A549, H1299, H1650	STAT3	miR-124 *via* inhibiting STAT3 could enhance radiation-induced apoptosis in NSCLC.	(22)
miR-125a-5p	Human	A549, H1299	NEDD9	miR-125a-5p *via* targeting NEDD9 could induce apoptosis in LUAD.	(23)
miR-125b	Human	A549	PI3K/Akt, GSK3β, Bax, Wnt, β-catenin	miR-125b through the PI3K/Akt/GSK3β pathway could regulate apoptosis in NSCLC.	(24)
miR-129-5p	–	A549, H1299	YWHAB	miR-129-5p *via* reducing YWHAB could induce apoptosis in LC.	(25)
miR-135a	Human	HBE, A549, H460, H1299	PI3K, Akt, GF-1, CD34, MVD	miR-135a *via* the IGF-1/PI3K/Akt pathway could promote apoptosis in NSCLC.	(26)
miR-139-5p	Human	A549	Hox-B2, P13k, Akt, Caspase-3	miR-139-5p by targeting Homeobox protein (Hox-B2) could promote apoptosis in NSCLC.	(27)
miR-140-5p	Human	A549	YES1, Bcl-2, Bax, Caspase-3	miR-140-5p *via* targeting YES1 could induce apoptosis in NSCLC.	(28)
miR-142	Human	BEAS-2B, A549, H1650	XIAP	miR-142 *via* targeting XIAP could promote apoptosis in LC.	(29)
miR-145	Human	A549	EGFR/PI3K/AKT, Bax	miR-145 by regulating the EGFR/PI3K/AKT pathway could induce apoptosis in NSCLC.	(30)
miR-145	Human	BEAS-2B, H1650, H1975, A549, H292	mTOR	miR-145 *via* negatively regulating the mTOR signaling pathway could influence apoptosis in NSCLC.	(31)
miR-146a-5p	GEO and TCGA databases	A549	TCSF	miR-146a-5p by targeting TCSF could influence apoptosis in NSCLC.	(32)
miR-155	–	A549, A549/R	Bax, Bcl-2, Cyto-Nrf2, Nucl-Nrf2, NQO1	miR-155 *via* activating Nrf2 could suppress apoptosis in LC.	(33)
miR-195-5p	Human	BEAS-2B, H1299, A549	CEP55, Bax, Bcl-2	miR-195-5p *via* targeting CEP55 could induce apoptosis in NSCLC.	(34)
miR-198	Human	A549, Calu-3	SHMT1, CDK1, Cyclin-D1/B1	miR-198 by targeting SHMT1 could enhance apoptosis in LUAD.	(35)
miR-210-3p	Human	BEAS-2B, A549, H358, H1650, H1299	SIN3A, Bcl-2, Caspase-3	miR-210-3p *via* targeting SIN3A could regulate apoptosis in NSCLC.	(36)
miR-216a-3p	Human	HBE, H1299 A549, H1975, PC9	COPB2, Bax, Bcl-2, Caspase-3	miR-216a-3p by targeting COPB2 could regulate apoptosis in LC.	(37)
miR-221	Human	BEAS-2B, A549, H322, H1299	HOTAIR	miR-221 *via* negative regulation of lncRNA HOTAIR could promote apoptosis in NSCLC.	(38)
miR-222-3p	Human	BEAS-2B, AH1299, SPC-A1, A549, 95D, 293T	BBC3	miR-222-3p *via* targeting PUMA (BBC3) could inhibit apoptosis in NSCLC.	(39)
miR-323-3p	–	A549, NCI-H3255, H1299	AKT, ERK, TMEFF2, Akt, ERK1/2	miR-323-3p by regulating AKT/ERK pathway *via* targeting transmembrane protein with EGF-like and 2 follistatin domain (TMEFF2) could inhibit apoptosis in NSCLC.	(40)
Hsa-miR-329	Human	A549, H1299	c-Met	Hsa-miR-329 *via* targeting oncogenic MET could promote apoptosis in NSCLC.	(41)
miR-377	Human	A549, H460, 95D, HCC82	CDK6	miR-377 by directly targeting CDK6 could promote apoptosis in NSCLC.	(42)
miR-379-5p	Human	BEAS-2B, A549, PG49, DMS-114	ARRB1, Bcl-2, Bax, Akt, P13K, Caspase-3	miR-379-5p *via* targeting β-rrestin-1 could promote apoptosis in NSCLC.	(43)
miR-384	Gene database (GEO)	BEAS-2B, A549, GLC82, MES-1, LTEP-s	COL10A1, Survivin, Bcl-2, Bax, Bcl-xl, Beclin1, LC3B	miR-384 *via* negative regulation of Collagen α-1(X) chain gene could induce apoptosis in NSCLC.	(44)
miR-425	Mice	BEAS-2B, A549, SK-MES-1	AMPH-1, Bcl-2, Caspase-3	miR-425 *via* targeting AMPH-1 could regulate apoptosis in NSCLC.	(45)
miR-484	Human	BEAS–2 B, A549, H1650, PC9	Apaf-1, PARP, Caspase-3	miR-484 *via* inhibiting Apaf-1 could suppress apoptosis in NSCLC	(46)
miR-503-3p	–	H292, H358, H1975	p21, CDK4	miR-503-3p *via* regulating p21 and CDK4 expression could induce apoptosis in LC.	(47)
miR-512-5p	Human	A549, H1299	p21	miR-512-5p through targeting p21 could induce apoptosis in NSCLC.	(48)
miR-512-5p	Human	16HBE, A549, H1299	ETS1, Bcl-2, Bax, Caspase-3/7, MMP-2/9	miR-512-5p *via* targeting ETS1 could induce apoptosis in NSCLC.	(49)
miR-513b	Human	A549, H460	HMGB3	miR-513b *via* targeting HMGB3 through regulation of the mTOR signaling pathway could regulate apoptosis in NSCLC.	(50)
miR-516a-3p	Human	16HBE, BEAS-2B, H1299, SPC-A1, A549	PTPRD	miR-516a-3p *via* targeting PTPRD could inhibit apoptosis in LUAD.	(51)
miR-593	Human	A549, H1299, H358, H1993	SLUG, Cyclin-D1, Akt, CDK4, CDK6, Bcl-2, Bax, E-cadherin, Vimentin	miR-593 *via* targeting SLUG−associated signaling pathways could promote apoptosis in NSCLC.	(52)
miR-608	Human	A549, HCC4006, 293T	TFAP4, Caspase-3	miR-608 *via* the inhibiting TFAP4 could promote apoptosis in NSCLC.	(53)
miR-654-3p	Human	A549	RASAL2, Bax, Bcl-2	miR-654-3p by targeting RASAL2 could promote apoptosis in NSCLC.	(54)
miR-875	Human	A549	SOCS2, Wnt, β-catenin	miR-875 by targeting SOCS2 could regulate apoptosis in NSCLC.	(55)
miR-1260b	Human	16HBE, H1299, SPCA1	Cyclin-D1, Bcl-2, p21, Caspase-3,	miR-1260b *via* targeting SOCS6 could regulate apoptosis in NSCLC.	(56)
miR-hsa-let-7g	Human	A549, H1944	HOXB1	miR-hsa-let-7g *via* targeting HOXB1 could inhibit apoptosis in LC.	(57)

Apoptosis-related miRNAs have been shown to influence survival of lung cancer patients. For instance, expression of miR-21 predicts lower OS of patients with NSCLC (12). Moreover, over-expression of miR-125b has been associated with poor prognosis in NSCLC (24).

## LncRNAs and Apoptosis in Lung Cancer

Expression of FER1L4 has been remarkably decreased in plasma and tissue samples of patients with NSCLC as well as related cell lines. Forced over-expression of this lncRNA has reduced cell proliferation, migratory aptitude and invasiveness. FER1L4 has been shown to up-regulate PTEN and p53 expressions, suppress AKT phosphorylation expression, therefore enhancing the fraction of apoptotic cells. Functionally, these effects are mediated through the PTEN/AKT/p53 pathway (58). On the other hand, expression of PCAT1 has been increased in NSCLC tissues and cell lines. *In vitro* studies have shown that PCAT1 stimulates cell proliferation and invasion while suppressing cell apoptosis. In addition, PCAT1 has been shown to interact with the RNA-binding protein DKC1. PCAT1 and DKC1 exert synergistic effects in NSCLC. They enhance activity of VEGF/AKT/Bcl-2/caspase9 pathway in these cells (59). WT1-AS is a down-regulated lncRNA in NSCLC cell lines which is shown to sponge miR-494-3p. Up-regulation of WT1-AS has increased apoptosis of lung cancer cells and attenuated progression of NSCLC through up-regulation of PTEN and subsequent inactivation of PI3K/AKT pathway (60). GACAT1 is another regulator of apoptosis which has been found to be up-regulated in NSCLC tissues in association with poor survival of patients. Functionally, GACAT1 enhances proliferation and cell cycle progression and inhibits apoptosis through sponging miR-422a and increasing expression of YY1 transcription factor (61). HOXC-AS2 is another up-regulated in NSCLC samples which increases proliferation, migration, and EMT, while suppressing apoptosis. HOXC13 has been identified as functional target of HOXC-AS2. Notably, HOXC-AS2 and HOXC13 can enhance expression of each other (62). Expression of SNHG1 has been found to be increased in NSCLC parallel with up-regulation of FRAT1. SNHG1 knock down has suppressed proliferation, increased cell apoptosis and precluded migration and invasiveness of these cells. Mechanistically, SNHG1 sponges miR-361-3p and to release FRAT1 from inhibitory effects of this miRNA (63). **Table 2** shows the role of lncRNAs in regulation of apoptosis in lung cancer.

**Table 2:** LncRNAs regulating apoptosis in lung cancer.

LncRNA	Sample	Cell line	Target/Pathway	Function	Ref
FER1L4	Human	–	PTEN, AKT, p53, Ki67, PCNA, MMP2/9, Bcl-2, Bax, Caspase-3/9	FER1L4 through the PTEN/AKT/p53 signaling pathway could promote cell apoptosis in NSCLC.	(58)
PCAT1	Rat	BEAS-2B, A549, A427, H460	VEGF, AKT, Bcl-2, Vimentin, N-cadherin, Caspase-3/8/9/12, DKC1, PARP, Cyclin-D, E-cadherin	PCAT1 through the VEGF/AKT/Bcl2/Caspase-9 pathway could regulate apoptosis in NSCLC cells.	(59)
WT1-AS	Human	16-HBE, A549, NCI-H1975, SK-MES-1	miR-494-3p, PTEN, PI3K, AKT, Bcl-2, Bax, Caspase-3, CDK2, Cyclin-E1	WT1-AS/miR-494-3p through the PTEN/PI3K/AKT Signaling Pathway could regulate apoptosis in NSCLC cells.	(60)
GACAT1	Human	NHBE, A549, H1299, H460, SK-MES-1	YY1, miR-422a	GACAT1 *via* sponging miR-422a could induce apoptosis in NSCLC cells.	(61)
HOXC-AS2	–	–	–	HOXC-AS2 *via* combining with the HOXC13 gene could mediate apoptosis in NSCLC.	(62)
SNHG1	Human	BEAS‐2B, H23, H1299	FRAT1	SNHG1 through the miR‐361‐3p/FRAT1 axis could influence cell apoptosis in NSCLC.	(63)
ASB16-AS1	Human	16HBE, A549, NCI-H266, NCI-H1299, SK-MES-1	p21, β-catenin, Cyclin-D1	ASB16-AS1 *via* activating the Wnt/β catenin signaling pathway could promote apoptosis of NSCLC.	(37)
PVT1	Human, mice	BEAS-2B, A549, PC-9, H157, H460	ITGB8, MEK, ERK	PVT1 *via* targeting miR-145-5p could regulate cell apoptosis in NSCLC.	(64)
LEF1-AS1	human	NCIH1993, NCI-H1581	miR-221, PTEN	LEF1-AS1 *via* regulating miR-221/PTEN Signaling could induce apoptosis in NSCLC.	(65)
MALAT1	Human, mice	BEAS-2B, H460, A549, H661, H358	miR-374b-5p, SRSF7	Knockdown of MALAT1 through miR-374b-5p/SRSF7 axis could regulate apoptosis in NSCLC.	(66)
MIR503HG	human	BEAS-2B, A549, NCI-H1299, NCI-H1975, NCI-H2170	Cyclin-D1/E, PCNA, p16, p21, Bcl-2, Bax, Caspase-3/9	MIR503HG *via* regulating miR-489-3p and miR-625-5p could promote apoptosis in NSCLC.	(67)
NEAT1	Human	BEAS-2B, A549, H292	SULF1, MAPK, Akt	NEAT1 *via* targeting has-miR-376b-3p/SULF1 axis could regulate apoptosis in NSCLC.	(25)
NEAT1	–	A549	miR-1224, KLF3	Knockdown of NEAT1 by sponging the miR-1224 could enhance the apoptosis in lung cancer	(68)
PRNCR1	Human	BEAS-2B, SPC-A1, A549	E-cadherin, N-cadherin, Vimentin, MTDH	Knockdown of PRNCR1 through sponging miR-126-5p could inhibit cell apoptosis in NSCLC treatment.	(69)
HCG11	Human, mice	A549, SPC-A1, H1299, H1650, H1975, PC-9	Caspase-3	HCG11 by Sponging miR-224-3p could promote apoptosis in NSCLC.	(70)
SNHG6	Human	BEAS-2B, A549, H460, H1299	Bcl-2, Bax, Caspase-3, RSF1	SNHG6 *via* regulating miR-490-3p/RSF1could inhibit apoptosis in NSCLC.	(71)
SNHG7	Human	BEAS-2B, H125, 95D, A594	FAIM2	SNHG7 *via* enhancing the FAIM2 expression could inhibit apoptosis in LC.	(72)
SNHG12	Human, mice	16-HBE, H1299, A549, H358, H1975, SPC-A1	miR-138	Knockdown of SNHG12 *via* Upregulating miR-138 could induce apoptosis in NSCLC.	(73)
SNHG14	Human, mice	PC9, PC9/GR	ABCB1, miR-206-3p	Knockdown of SNHG14 by sponging miR-206-3p *via* upregulating ABCB1 could induce apoptosis in NSCLC.	(13)
SNHG20	Human, mice	A549, H32, H1299, GLC-82, SPC-A1	ZEB2, RUNX2	Knockdown of SNHG20 by acting as a miR-154 sponge could promote apoptosis in NSCLC.	(45)
	Human	16HBE, A549, H1299	E2F3, P13k, Akt	Knockdown of SNHG20 *via* regulating miR-2467-3p/E2F3 could induce apoptosis in NSCLC.	(74)
	Human	16HBE, A549, NCI-H520, H1299	TCF4, LEF1, Wnt/β-catenin	SNHG20 *via* Wnt/β-catenin signaling pathway by targeting miR-197 could inhibit the apoptosis of NSCLC cells.	(75)
SNHG20	Human	16HEB, A549, H1299	DDX49, miR-342	Knockdown of SNHG20 by sponging miR-342 and upregulating DDX49 could promote cell apoptosis in lung adenocarcinoma.	(76)
HAND2-AS1	Human	BEAS-2B, NCI-H23, NCI-H522	PI3K, Akt	HAND2-AS1 *via* inactivating PI3K/Akt pathway could promote cell apoptosis in NSCLC.	(77)
PANDAR	Human	16HBE, L78, PC9, 95D, NCI-H460, A549	Beclin-1, LC3-I, LC3-II	PANDAR by upregulation of BECN1 expression *via* activating autophagy and apoptosis pathways could inhibit the development of lung cancer.	(14)
LINC00460	Murine	A549	miR-539	LINC00460 *via* targeting miR-539 could inhibit apoptosis in NSCLC.	(78)
ATB	–	NCI-H838, BEAS-2B	Bcl-2, Caspase-3, CytC	ATB *via* suppressing the expression of miR-200a and up-regulating the expression of β-catenin could promote apoptosis in NSCLC.	(79)
AWPPH	Human	WI-38, NCI-H23, NCI-H522	Wnt, β-catenin	AWPPH *via* activating the Wnt/β−catenin signaling pathway could inhibit apoptosis in NSCLC.	(80)
DLX6-AS1	Human, mice	16HBE, H1975, A549	PRR11	Knockdown of DLX6-AS1 *via* downregulating PRR11 expression and upregulating miR-144 could promote apoptosis in NSCLC.	(53)
BANCR	Human, mice	A549, SPC-A1, H1299, H1650, H1975, PC-9	Bcl-2, Bax	Overexpression of BANCR could increase apoptotic level.	(81)
TSLNC8	Human	HBE, A549, H441, H1975	CDK2, Cyclin-E1, p21, MMP9, Bcl-2, Bax, Caspase-3	TSLNC8 *via* targeting the IL-6/STAT3/HIF-1α signaling pathway could accelerate apoptosis in NSCLC.	(82)
AFAP1-AS1	Human, mice	BEAS-2B, H1975, PC-9, A549, SPCA-1	RRM2, EGFR, Akt	AFAP1-AS1 *via* Competitively upregulating RRM2 by sponging miR-139-5p could reduce apoptosis in NSCLC.	(83)
PCAT-1	Human	16HBE, H1299, SK‐MES‐1	PCAT‐1, LRIG2	Knockdown of PCAT‐1 *via* regulating miR‐149‐5p/LRIG2 axis could induce apoptosis promotion in NSCLC.	(84)
HULC	Human	NCI-H23, NCI-H522	PI3K, Akt, SPHK1	HULC *via* upregulating sphingosine kinase 1(SPHK1) and its downstream PI3K/Akt pathway could inhibit apoptosis in NSCLC.	(85)
EPB41L4A-AS2	Human	16HBE, SK-MES-1, HCC827, A549, NCI-H1975	PCNA	Overexpression of EPB41L4A-AS2 could promote apoptosis in NSCLC.	(86)
NBAT-1	Human	A549	RAC1	NBAT-1 by downregulating RAC1 could promote Cell Apoptosis in NSCLC.	(87)
PICART1	Human	BEAS-2B, A549, SPC-A-1, NCI-H358, NCI-H1975, HCI-H292	Twist1, MMP2/9, E-cadherin, Cyclin D1, p21, Bcl-2, Bax, Caspase-3, STAT3, JAK2	PICART1 *via* inhibiting JAK2/STAT3 signaling could promote apoptosis in NSCLC.	(30)
CASC2	Human	16HBE, A549, H1299	p62,Atg-5, LC3-I, LC3-II, LC3-II/I, TRIM16,	CASC2 by regulating the miR-214/TRIM16 axis could promote apoptosis in NSCLC.	(88)
LINC00961	Database	A549, H226	PCNA, Bax	LINC00961 *via* regulating PCNA could induce cell apoptosis in NSCLC.	(89)
LINC02418		16HBE, A549, PC-9	miR-4677-3p, SEC61G	LINC02418 *via* regulating miR-4677-3p/SEC61G could regulate apoptosis in NSCLC.	(90)
00312	Human, mice	A549, SPC-A1, H1299, H1975, PC9, H1703, H520, SK-MES	HOXA5	lncRNA00312 *via* inhibiting HOXA5 could promote apoptosis in NSCLC.	(91)
TRPM2-AS	Human	A549, H1299	SHC1	Knockdown of TRPM2-AS could increase cell apoptosis in NSCLC.	(92)
MEG3	Human, mice	A549	miR-205-5p, LRP1, p53,p21, Caspase-3	MEG3 through the miR-205-5p/LRP1 pathway could regulate apoptosis in NSCLC.	(46)
TUG1	Human	16HBE, A549, SPC-A1, PC-9, H1299, H1975	EZH2, Bax, BCL2, BCL2A1, PARP2, BIRC3, MCL1, BAK1, CASP9, CASP3	TUG1 through the epigenetic silencing of BAX could suppress apoptosis in LUAD.	(21)
LINC00857	Human	BEAS-2B, H1229, H838	miR-1179, SPAG5	LINC00857 *via* targeting the miR-1179/SPAG5 axis could regulate apoptosis in lung adenocarcinoma.	(93)
LINC00472	Human	BEAS-2B, A549, H1299, H460, H446	miR-24-3p, DEDD	LINC00472 by regulating miR-24-3p/DEDD could promote Apoptosis of LUAD,	(94)
AFAP1-AS1	Murine	A549	miR-545-3p, HDGF	Knockdown of AFAP1-AS1 by regulating the miR-545-3p/HDGF axis could promote apoptosis in lung cancer.	(95)
ZEB2-AS1	Human	MRC-5, 95D, H-125, A549, NCI-H292, H1975	Bcl-2, Bax, Caspase-3/9	In A549 and NCI−H292 cells, knockdown of ZEB2-AS1 could inhibit cell proliferation, while in H-125 and H1975 cells overexpression of ZEB2-AS1 could inhibit cell apoptosis.	(96)
NORAD	Human	H460, H1299, A549, HBE, SCLC-21H	ADAM19, miR-30a-5p	Knockdown of NORAD *via* regulating miR-30a-5p/ADAM19 could promote cell apoptosis in LC	(97)

Among lncRNAs which regulate apoptosis in lung cancer cells, over-expression of LINC00460, AWAPPH, SNHG20, HULC, ZEB2-AS1 and TRPM2-AS has been associated with poor prognosis of patients, while EPB41L4A-AS2 has the opposite effect (**Table 3**).

**Table 3:** Prognostic role of apoptosis-related lncRNAs in lung cancer.

Sample	Kaplan-Meier Analysis	Ref
483 NSCLC tissues and 347 paracancerous tissues were TCGA	Higher expression of LINC00460 was associated with poor prognosis of NSCLC	(78)
88 patients with NSCLC including 56 males and 32 females, aged 23–71 years (average age, 46 ± 8.9 years)	Higher expression of AWPPH was associated with poor prognosis of NSCLC	(80)
42 paired NSCLC tumor and adjacent normal tissue samples from Wenzhou Central Hospital	Higher expression of SNHG20 was associated with a poor prognosis of NSCLC	(45)
102 patients (61 males and 51 females) with NSCLC aged from 21-76 years, with an average age of 48 ± 7.7 years.	Higher expression of HULC was associated with poor prognosis of NSCLC	(85)
100 lung cancer tissues and their adjacent non-cancerous tissues from patients (67 males and 33 females; mean age, 62; age range, 48–79)	Higher expression of ZEB2-AS1was associated with higher proliferation of NSCLC.	(96)
56 NSCLC and adjacent tissues	Lower levels of EPB41L4A-AS2 was associated with poor prognosis in NSCLC	(86)
60 NSCLC patients	Higher expression of TRPM2-AS was associated with poor prognosis in NSCLC	(92)

## ncRNAs, Cell Apoptosis and Immunotherapy

Since immunotherapy has an emerging role in the treatment of lung cancer (98), identification of the role of ncRNAs in immune regulation and response of lung cancer to immunotherapy is important. A number of apoptosis-regulating ncRNAs have essential roles in this regard. For instance, miR-155 and miR-17~ 92 are involved in differentiation regulatory T cells (Tregs) and their function (99). miR-21 and miR-26 through down-regulation of TAP1 and reduction in expression of HLA class I antigens affect response to immunotherapies (100). miR-138, miR-155, miR-34 and miR-146a have been found to affect immune checkpoints (101). MALAT1 is an lncRNA which is possibly involved in the immunotherapy resistance through induction of immunosuppressive phenotypes in stem cells (102). NEAT1 can affect response to immunotherapy through modulation of miR-155/Tim-3 (103). The exact roles of these ncRNAs in conferring resistance to immunotherapeutic approaches have not been elucidated in lung cancer; yet based on the results obtained from similar studies in other cancer types, these ncRNAs are expected to simultaneously affect apoptosis and response to immunotherapy in lung cancer.

## Discussion

**Figure 3:**
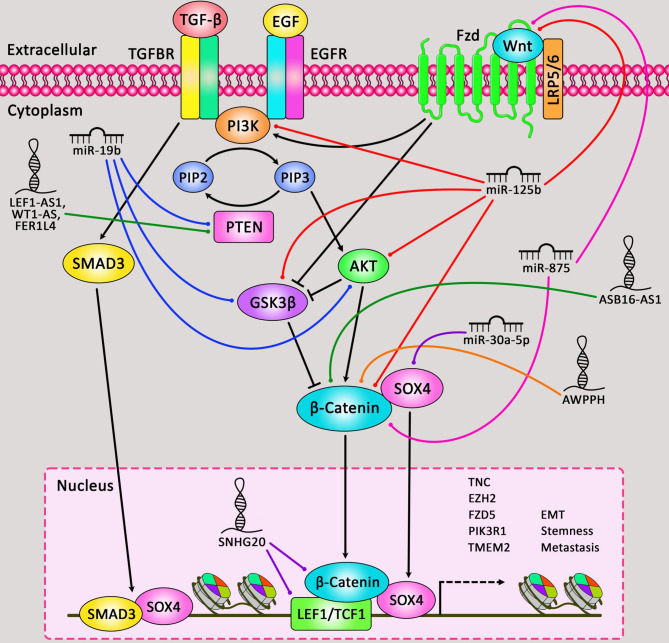
A schematic summary of the role of miRNAs and lncRNAs in regulating apoptosis cascade in lung cancer *via* Wnt/β-catenin pathway. Accumulating evidence has delineated that apoptotic cells are negative for β-catenin. This indicates that the Wnt/β-catenin signaling cascade could be inactive in apoptotic cells. Whilst, β-catenin is expressed in the membrane, cytoplasm, and nucleus of non-apoptotic epithelial cells around these apoptotic cells. Therefore, Wnt/β-catenin signaling cascade could be activated in non-apoptotic epithelial cells *via* apoptotic cells (104). As an illustration, downregulation of miR-125b could play an effective role in inhibiting expression of p-Akt, p-GSK3β, Wnt, and β-catenin, and could promote caspase-3 activity and Bax protein expression in human non-small cell lung cancer. Thereby, this could lead to suppressing the proliferation and triggering the apoptosis of tumor cells (24). Furthermore, another study have illustrated that upregulation of lncRNA SNHG20 could have a crucial part in elevating the proliferation and suppressing the apoptosis of NSCLC cells through targeting miR-197 *via* regulating the Wnt/β-catenin signaling cascade. Downregulation of this lncRNA could result in remarkable reduction of TCF and LEF1 expression in the Wnt/β-catenin pathway (75).

Cell apoptosis, as one of the major dysregulated processes in the carcinogenesis of lung cancer has been shown to be regulated by ncRNAs. In the current review, we have explained the impact of miRNAs and lncRNAs on apoptosis in lung cancer. These ncRNAs interact with PI3K/Akt, NF-κB, Wnt/β-catenin, EGFR, TGF-β and other cancer-related pathways. Therefore, they not only regulate apoptosis, but also influence other aspects of lung carcinogenesis. **Figure 3** depicts the role of ncRNAs in modulating apoptosis through Wnt/β-catenin cascade in human lung cancer.

Manipulation of expression of apoptosis-regulating lncRNAs and miRNAs represent a strategy for combating carcinogenesis as well as resistance to chemo/radiotherapy. Some of the apoptosis-regulating miRNAs/lncRNAs have been shown to influence prognosis of lung cancer. The observed correlation between their expression and patients’ survival is due to their impact on disease progression as well as response of patients to EGFR inhibitors and chemotherapeutic agents. EMT is another important feature of lung cancer cells which is regulated by a number of apoptosis-regulating miRNAs/lncRNAs indicating the intercalation between cancer-related processes.

An acknowledged route of function of lncRNAs in the regulation of apoptosis in lung cancer is their impact on expression of miRNAs. In fact, they can sequester miRNAs and release miRNA targets from their inhibitory effects. WT1-AS/miR-494-3p, LEF1-AS1/miR-221, NEAT1/miR-1224, SNHG12/miR-138, LINC02418/miR-4677-3p, MEG3/miR-205-5p, LINC00857/miR-1179, LINC00472/miR-24-3p, AFAP1-AS1/miR-24-3p and NORAD/miR-30a-5p are examples of lncRNAs/miRNAs interactions with verified roles in the control of lung cancer cells apoptosis.

Based on the importance of apoptotic pathways in determination of response of lung cancer patients to conventional as well as targeted therapies, identification of the impacts of lncRNAs/miRNAs on apoptosis and prior profiling of these ncRNAs in clinical samples would help in prediction of response of patients to each therapeutic regimen and design of personalized treatment strategies. The advent of high throughput sequencing strategies has facilitated conduction of this approach in the clinical settings.

Finally, the possibility of lncRNAs/miRNAs tracing in the peripheral blood of patients has opened a new opportunity for early detection of emergence of resistance to conventional or targeted therapies and modulation of therapeutic regimens to enhance the survival of affected individuals.

## Author Contributions

MT and SG-F wrote the draft and revised it. HS, AA, JM, and MM collected the data, designed the tables and figures. All authors contributed to the article and approved the submitted version.

## Conflict of Interest

The authors declare that the research was conducted in the absence of any commercial or financial relationships that could be construed as a potential conflict of interest.
